# Rapeseed Meal as an Alternative Protein Source in Fish Feed and Its Impact on Growth Parameters, Digestive Tract, and Gut Microbiota

**DOI:** 10.3390/ani15091264

**Published:** 2025-04-29

**Authors:** Wnęk-Auguścik Karolina, Nasiłowska Justyna

**Affiliations:** 1 Department of Animal Environment Biology, Institute of Animal Sciences, Warsaw University of Life Sciences—SGGW, 8 Ciszewskiego Street, 02-786 Warsaw, Poland; 2 Department of Microbiology, Prof. Waclaw Dabrowski Institute of Agricultural and Food Biotechnology—State Research Institute, 36 Rakowiecka Street, 02-532 Warsaw, Poland; justyna.nasilowska@ibprs.pl

**Keywords:** fish, feed, rapeseed meal, growth parameters, digestive tract, gut microbiota

## Abstract

As traditional protein sources such as soybean and fish meal become increasingly limited, there is growing interest in alternative protein sources, such as rapeseed meal, in aquaculture. This review offers an updated overview of rapeseed meal in fish diets, highlighting its nutritional value and impact on growth and the digestive tract. It also examines the effect of rapeseed meal supplementation on intestinal microflora, taking into account the latest trends in animal breeding focused on microbiota composition. While challenges exist in incorporating rapeseed meal into fish diets, its inclusion holds potential for improving sustainability and efficiency in aquaculture. However, its effects may vary depending on fish species, rearing conditions, and the level of inclusion in the diet. Based on this analysis, further research is recommended to explore the practical use of rapeseed meal as a reliable protein source in fish nutrition. Future studies should prioritize fish welfare and health, with an emphasis on natural methods, such as fermentation, to enhance the nutritional value of rapeseed meal. Consideration should also be given to incorporating activities, such as phytase supplementation, to enhance the use of rapeseed meal in fish feed.

## Simple Summary

Aquaculture is among the fastest-growing sectors in agriculture. Traditionally, fish feeds are based on fishmeal and soybean meal as primary sources of protein, both of which are essential for the proper growth and development of fish. However, due to the limited availability and rising costs of these ingredients, there is increasing interest in alternative, plant-based protein sources such as rapeseed meal. Rapeseed meal, a by-product of the oil extraction process, is high in protein and presents a promising alternative. Given the diversity of species in aquaculture, the effects of including rapeseed meal in fish diets can vary considerably. Numerous studies have explored its potential as a feed ingredient, examining how it influences growth performance, digestive tract health, and gut microbiota. These effects often depend on both the level of inclusion and the duration of its use in the diet. This paper reviews recent findings on the application of rapeseed meal in aquafeeds and highlights key areas for future research, with particular emphasis on its implications for fish health.

## 1. Introduction

Aquaculture is the fastest-growing sector of animal production, and the biomass of farmed fish exceeds their capture from natural environments [1]. Fish are a valuable source of protein, essential unsaturated fatty acids, minerals, and vitamins for humans. The demand for aquaculture products is steadily increasing, as fish and their products are considered functional and health-promoting foods [2]. As ectothermic animals, fish use proportionally more energy for growth than endothermic animals, and the lack of necessity to convert ammonia into urea allows them to feed on protein-rich food. Aquaculture is an element of sustainable agricultural development. Fish farming, due to its lower CO_2_ emissions compared with land animal farming, is more environmentally friendly and also contributes to water retention and the increase of biodiversity by creating habitats for various organisms [3].

Protein remains the costliest ingredient in fish feeds. Fish meal contains between 60% and 72% crude protein by weight and is commonly used as its primary source due to its content of essential amino acids and highly absorbable minerals, including phosphorus [4]. Furthermore, this product increases the palatability and digestibility of the feed [5]. It is obtained both from the by-products of the processing of various fish species and from fisheries specifically conducted for this purpose [6]. The use of fish meal has been associated with high prices for some time, partly due to the increase in global fish production for human consumption [7], rising fish meal production costs, and the limitation of its resources [8] which is also due to the sensitivity of fish availability to climate change [5]. Taking these conditions into consideration, the use of fish meal in aquaculture feeds should be reduced. A reduction in the fish meal content in fish nutrition can be implemented without affecting overall production efficiency [7], and the search for alternative protein sources remains a constant priority for the industry [9]. Therefore, animal-derived protein products in feeds are often replaced by cheaper plant-based alternatives, primarily soy and its derivatives.

More than half of global soybean production is used as animal feed, meaning that most livestock obtain protein primarily from soy and its derivatives. Soy is characterized by its high protein content and a balanced amino acid profile [5]; in turn, soybean meal is a raw material that is significantly cheaper than fish meal [10]. In some cases, soy-based feeds allow for the complete replacement of fish meal, such as with red sea bream (*Pargus major*) [11] or Nile tilapia (*Oreochromis niloticus*) [12]. However, in the case of many fish species, such as Atlantic cod (*Gadus morhua*) [13], Japanese sea bass (*Lateolabrax japonicus*) [10], or largemouth bass (*Micropterus salmoides*) [14], using soybean meal in feed allows for only the partial replacement of fish meal. Moreover, soy products contain antinutritional substances such as protease inhibitors, phytates, saponins, lectins, and oligosaccharides, which may reduce the digestibility of polypeptides, the bioavailability of mineral nutrients, and feed intake [9].

The growth of the human population and its increasing demand for meat, along with the dynamic development of intensive animal farming, primarily in Asia but also on other continents, has led to a rise in global soybean production (from 20 million to 350 million tons annually over the past 50 years) [15]. Due to environmental requirements, soy is mainly grown in the USA, Brazil, and Argentina. For many countries, due to climatic conditions, importation from abroad is necessary [16,17]. In recent years, consumers have become increasingly aware of the issues related to animal products, as well as animal welfare and nutrition concerns. As a result, some farmers and consumers prefer to use feeds without genetically modified (GMO) plants. However, the availability of non-genetically modified soy is still too low to meet these demands [18]. The majority of global soybean crops, and almost all of them in the Americas (94–97%), consist of genetically modified plants [19]. Additionally, many countries, including those in Europe, are not ready to produce and provide soybean protein in animal feed. Therefore, EU countries have been advocating for the diversification of protein sources in the feed market by increasing the use of domestic plant protein sources [20,21].

## 2. Rapeseed Meal as an Alternative Source of Protein in Fish Feed

At the beginning of the 21st century, global rapeseed (*Brassica napus* L.) production experienced dynamic growth, increasing at an annual rate of 4% [22,23]. This growth is driven by the rising consumption of vegetable oils and the increasing demand for protein in animal feed. Moreover, rapeseed cultivation offers high profitability and can be grown across a broad range of temperatures and humidity levels [24]. Global rapeseed production totals approximately 70 million tons of raw grain, with the majority sourced from Asia, Europe, and North America [25]. The top producers include China (14.7 million tons), Canada (13.7 million tons), and India (10.2 million tons) [25]. In Europe, Germany (1166.80 ha) and France (1343.78 ha) are the leading countries in rapeseed cultivation [26].

Rapeseed seeds contain 40–50% fat, making this plant one of the most important oilseed crops [22,27,28]. Post-extraction rapeseed meal is a by-product of oil extraction from rapeseed and biodiesel production. Additionally, compared with soybean meal, it is significantly cheaper [29,30]. It is an excellent source of protein, with a content ranging from 30% to as much as 40%, with 2% fat content on a dry matter basis and fiber levels from 33% to 40% [31]. Furthermore, it has a well-balanced amino acid profile and provides essential amino acids such as methionine and threonine [32,33,34]. The total carbohydrate content is 9.3%, with sucrose being the main carbohydrate [35]. Sucrose and fat are the basic energy components of rapeseed meal [36]. Compared with other plant-based protein sources, rapeseed meal shows a high level of choline and niacin [28]. Other vitamins present in rapeseed meal include biotin, riboflavin, and thiamine. This meal also has a high content of calcium, phosphorus, selenium, iron, and manganese [28]. The ash content in rapeseed protein products is lower than in fish meal, which reduces the risk of decreasing the digestibility of proteins, lipids, and dry matter in the feed [37]. The detailed chemical composition of rapeseed and soybean meal is presented in Table 1.

However, some reports indicate that rapeseed protein is less digestible than other plant and animal protein sources. Additionally, rapeseed products contain antinutritional compounds—such as glucosinolates (50–100 μmol/g dry matter), erucic acid (25–45% in the oil), and phytic acid (1–5% in dry matter) [40]. These substances may negatively affect nutrient absorption in the feed, which in turn can impact morphometric, breeding, and health indicators [41]. Little is known about the metabolism of antinutritional substances in fish compared to other animal groups. Erucic acid, for example, has been associated with fat deposition in the liver and kidneys, as well as heart muscle damage [42]. According to [43], glucosinolates have been shown to decrease feed palatability, reduce fish growth, and negatively affect overall production. Moreover, fish are at risk from the breakdown products of these compounds, which can cause liver cell damage, disrupt thyroid metabolism, and may have neurotoxic effects [37]. The antinutritional factors in rapeseed can be reduced using various techniques, including physical, chemical, and biological methods, and breeding improved plant varieties. Physical methods include, for example, steam heating, roasting, microwaving, and treatment. Acid and alkaline degradation, metal salts degradation, and solvent extractions constitute the chemical methods. These processes, when extended over time, cause physical and chemical changes to the proteins, which in turn impact their nutritional value [38]. Biological methods usually include microbial fermentation, which is an effective method for reducing antinutritional compounds in rapeseed meal while enhancing its nutritional value. Research has shown that fermentation with selected bacterial strains can boost the content of small peptides and improve the meal’s palatability. However, the effectiveness of this approach can vary depending on the microorganisms involved, as well as the specific conditions and duration of the fermentation process [39,40]. To improve the quality of rapeseed-based feeds, so-called canola that are “double-zero” varieties (“00”) are currently used. The development of these varieties has revolutionized and expanded the rapeseed market [34,36,44,45]. “Double-zero” varieties are characterized by their significantly reduced concentrations of two key antinutritional substances: erucic acid (<2% of the total fatty acid content) and glucosinolates (<30 µmol/g dry matter of defatted meal) [46,47,48]. Rapeseed is known for having the highest concentration of phenolic compounds among the oilseeds [36]. These compounds include phenolic acids and condensed tannins, which are responsible for the plant’s bitter taste [49]. Although they are present in small quantities in the seeds, rapeseed meal typically contains less than 1.5% of these compounds, as most are removed during the oil extraction process [36]. Additionally, it is noteworthy that rapeseed meal contains fewer antinutritional compounds belonging to the phytoestrogen group compared with soybean meal [50].

Phosphorus in rapeseed meal is predominantly present as phytates, which are salts of phytic acid. These phytates can bind minerals such as calcium, magnesium, and potassium, forming complexes that are less digestible for monogastric animals. The ruminant animals and fish have minimal phytase activity, an enzyme required for the degradation of phytates [51]. The exact role of phytic acid in animal nutrition is not fully understood, but it is considered an antinutritional factor [52]. In livestock nutrition, the addition of exogenous enzymes is a commonly used method to improve the utilization of nutrients from feed. A significant improvement in the nutritive value of rapeseed meal has been observed with the use of phytase [52]. Supplementing a diet containing rapeseed cake with phytase increases phosphorus digestibility and significantly improves production parameters in monogastric animals [53,54]. Phytase is also used in fish feed, and studies demonstrate that dietary phytase has significant potential to improve the nutritional value of rapeseed products, for example, for rainbow trout [55].

The demand for affordable, nutritious, and non-genetically modified feed is steadily increasing, prompting more producers to explore alternatives to post-extraction soybean meal [56]. Rapeseed is widely available on a global scale and offers one of the most balanced essential amino acid compositions among plant-based protein sources. Additionally, rapeseed cultivation is characterized by low production costs and ecological and economic benefits in agriculture. Rapeseed meal holds significant potential to replace soybean meal in various types of animal feed. Today, both rapeseed seeds and post-extraction meal are among the most important substitutes for post-extraction soybean meal in livestock feed [33]. Due to its valuable protein content, which offers a beneficial amino acid profile similar to that of soybean meal, rapeseed meal is currently being successfully utilized in pig and cattle farming [57,58,59]. Following post-extraction soybean meal, rapeseed meal is the second most commonly used plant-based ingredient in aquaculture feed [60]. Research from various studies on the use of rapeseed products in fish nutrition indicates that rapeseed meal is an effective partial (10–66%) substitute for fish meal in the diets of various fish species [61,62,63], while rapeseed protein concentrate can replace animal protein by 25–100% [64,65,66].

## 3. The Influence of the Use of Rapeseed Meal in Fish Diets on Growth Parameters

The successful substitution of fish meal with plant-based protein alternatives in fish feed is an important direction for the development of aquaculture [67,68]. However, there are reports highlighting the negative aspects of using rapeseed meal in fish diets. Rapeseed meal has a high content of sulfur-containing amino acids (AAs) but is low in lysine. Therefore, diets with a high supplementation of this meal may not be as nutrient-efficient as fish meal-based diets [69], which could, in turn, reduce growth parameters in some species. Many discrepancies exist in scientific reports regarding the results obtained. However, it has been suggested that these discrepancies could be attributed to differences in feed nutritional composition, ingredient quality, and processing methods, as well as environmental factors, fish age, or genotype [70]. A detailed summary of the current findings on rapeseed meal supplementation in fish feed is presented in Table 2.

## 4. The Condition of the Digestive Tract After Supplementation with Rapeseed Meal

Plant ingredients in fish feeds can influence metabolism, the condition of the digestive tract, and overall health, as well as the performance and quality of fish production [83]. The intestines, along with the liver, are crucial organs responsible for digestion and nutrient absorption from feed [84,85]. These organs can be regarded as indicators, and their condition should be monitored to assess the impact of nutritional components and the fish’s nutritional status [86]. Histological studies of the digestive tract are particularly important, as they represent one of the potential pathways for the entry of pathogenic microorganisms, which is significant since dietary protein sources can affect the intestinal integrity of fish [87,88].

Despite the potential for using plant materials as a protein source, their inclusion in the diets of fish, particularly carnivorous species, is limited due to the adverse effects, primarily the occurrence of inflammatory conditions in the intestinal epithelium [89]. Diets of Atlantic salmon (*Salmo salar*) based on plant proteins compromised the intestinal barrier, increased the volumetric density of goblet cells, caused signs of intestinal inflammation, and reduced villus height [90]. A marked decrease in microvilli height, swelling of the lamina propria and submucosa, an increase in the number of goblet cells, and loss of the mucosal fold architecture were also observed by other authors in salmonid fish [91,92,93]. High levels of rapeseed meal in the feed of the large yellow croaker (*Larimichthys crocea*), ranging from 36% to 60%, caused disorganization and reduced villus twisting in the middle part of the intestine, which could reduce the absorptive surface area and cause digestive dysfunction. These effects became more pronounced as the level of rapeseed meal supplementation increased [94]. In contrast, in barramundi (*Lates calcarifer*) 30% inclusion of rapeseed meal in feed did not induce inflammatory changes in the lamina propria of the distal intestine, and the histological results were comparable to those obtained with fish meal as a protein source [95]. In gilthead seabream (*Sparus aurata*), when 20% rapeseed meal was included in the diet, a tendency for an increase in villus height in the foregut was observed. The researchers suggested that this was likely related to enhanced absorptive functions [96].

Omnivorous fish usually have a greater tolerance for an increased proportion of plant protein, which is due to the different digestive enzyme activities, intestinal morphology, and intestinal microbiota compared with carnivorous fish [97,98]. Iqbal et al. [2] observed increased variability in the morphology of the mucosal and submucosal layers, villus length, lamina propria width, and vacuolization in Nile tilapia (*Oreochromis niloticus*) with a high supplementation of rapeseed meal. However, inflammatory conditions, increased numbers of dead cells, and total degeneration of tissue walls and villi in the intestine appeared only at 100% replacement of fish meal and soybean meal with rapeseed meal. Despite this, the survival rate was similar among all groups, indicating that rapeseed meal supplementation did not increase fish mortality. In the study by [62] on the same species, significant increases in intestinal villus length were observed in groups supplemented with rapeseed meal compared with the control groups. The same authors also noted that replacing part of the fish meal with rapeseed meal increased the number of goblet cells in all experimental groups compared with the control groups. An increase in the number of goblet cells in the small intestine may indicate a rise in mucin secretion [99] that may affect nutrient absorption. After 8 weeks of feeding juvenile goldfish (*Carassius auratus gibelio × Cyprinus carpio*) a diet containing 50% rapeseed meal, mild histological changes were observed in the anterior part of the intestine, without massive lymphocytic infiltration or vacuolization. These changes included a shortening of the intestinal folds, a reduction in the thickness of the muscular layer, an increase in goblet cell number, and enhanced mucus secretion [75]. In the study by [80], the length of intestinal villi, villus width, and muscle thickness decreased in black carp (*Mylopharyngodon piceus*) when fish meal was replaced with rapeseed meal at levels exceeding 10%.

Studies have reported that nitriles formed from glucosinolates may contribute to the impairment of liver and kidney functions [100]. In the experiment conducted by [82], fat cells were observed in the livers of grass carp (*Ctenopharyngodon idella*) fed rapeseed meal. Hepatopancreatic lipid content increased as the level of dietary rapeseed meal increased. In the groups where rapeseed was added at 32% and 48%, both nucleus migration and lipid vacuoles in the cytoplasm were observed. In the group receiving 64% rapeseed meal, parenchymal necrosis, irregularly shaped hepatocytes, and heavy condensation of the hepatocyte cytoplasm with a loss of intracytoplasmic vacuoles were noted. The lipid deposition in the liver is partly due to the nutritional deficiencies of rapeseed meal [101], but supplementation with methionine, choline, betaine, and lecithin can reduce lipid accumulation in the liver and promote the transport of lipids from the liver to extra-hepatic tissues [102].

## 5. Diversity of Gut Microbiota After Supplementation with Rapeseed Meal

Protein, as a primary ingredient in fish feeds, plays a central role in fish development and maintaining the integrity of the intestinal mucosa while providing nutritional support to gut microorganisms [103,104]. The intestinal microbiota also changes when plant ingredients are included in the diet [91,92,105,106]. However, rapeseed meal and its impact on fish gut microbiota has received so far relatively little attention.

Rapeseed meal contains fiber, including cellulose and hemicellulose, which are indigestible by the fish but serve as a food source for gut microorganisms [107]. Cellulolytic bacteria in the fish’s intestines can break down these polysaccharides into smaller molecules, which affects the microbiota composition and promotes the growth of bacteria capable of fermenting fiber, such as *Erysipelatoclostridium*, *Leptotrichi*, and *Anaerorhabdus furcosa* [108]. Additionally, most bacteria belonging to the genera *Lactobacillus*, *Bifidobacterium*, and *Cetobacterium* may be promoted, while other bacteria from the *Firmicutes* or *Proteobacteria* groups may be reduced or altered in response to a fiber-rich diet [109,110,111]. The non-starch polysaccharides in rapeseed meal delay digesta passage in the intestinal tract [112]. This results in decreased oxygen tension in the intestine and favors the development of anaerobic microflora [113].

Recent evidence shows that some members of lactic acid bacteria, notably the genera *Lactococcus* and *Carnobacterium*, are important transient or permanent inhabitants of the gastrointestinal tract of fish [114,115]. These beneficial gut bacteria are known to have antipathogenic effects [116,117], e.g., they contribute to the accelerated growth of grass carp (*Ctenopharyngodon idella*) [108,118]. However, reports indicate that in some fish species, such as rainbow trout (*Oncorhynchus mykiss*) and marbled eel (*Anguilla marmorata*), microbiota differences were not significantly associated with fish growth rate [119].

Glucosinolates present in rapeseed meal exhibit antibacterial properties, and their presence in fish diets can also alter the composition of gut microbiota, reducing the number of bacteria that are sensitive to these compounds. On the other hand, glucosinolates may induce oxidative stress or alter the bacterial flora toward microorganisms resistant to these compounds [120,121]. It has been proved that the high protein content in rapeseed meal can influence the growth of bacteria that break down proteins and amino acid reactions releasing numerous metabolites in the response to increased amino acid availability. The production of metabolites such as ammonia or biogenic amines may also negatively affect fish health [122]. Moreover, the inclusion of rapeseed meal in the diet of Nile tilapia (*Oreochromis niloticus*) and African catfish (*Clarias gariepinus*) [123,124,125] can alter the profile of the intestinal metabolism, including the production of short-chain fatty acids (SCFAs). The production of these acids in the digestive tract may lower the pH, which could disturb the normal microbiota present in the gut [75]. SCFAs, such as butyric, propionic, and acetic acids, are products of fiber fermentation by secondary fermentative bacteria from the *Paludibacteraceae* family [126]. On the other hand, the results of many studies confirm that these acids also promote a healthy microbiota and maintain the integrity of the intestinal barrier. A wide range of organic acids and short-chain fatty acids (SCFAs) have been tested in the diets of aquatic species [127]. These compounds resulted in a significant improvement in fish performance, increased the bioavailability of minerals, and potentiated fish health status, antioxidant activity, and immune responses [127,128,129]. Examples include the effects of citric acid in Beluga (*Huso*) [130], propionic acid salts in Zebrafish (*Danio rerio*) [131,132], and malic acid in Nile tilapia (*Oreochromis niloticus*) [133]. Interestingly, increased leucine intake in a plant-based protein diet has been correlated with an increase in the pyruvate fermentation pathways leading to butanoate and propanoate production [122].

The impact of rapeseed meal on fish gut microbiota depends on other dietary components, such as the amounts of fats, carbohydrates, vitamins, and minerals [134]. Nutrient availability may stimulate goblet cells in the intestinal mucosa to secrete mucin, which in turn influences the abundance of certain gut bacteria (e.g., *Bacteroides thetaiotaomicron*, *Akkermansia muciniphila*, and *Barnesiella intestinihominis*) that can degrade mucin glycans [135]. In some instances, this may allow opportunistic bacteria to pass through the mucus barrier, and, upon reaching the epithelial cells, may activate an immune response [136]. A varied diet can support beneficial changes in the gut microbiota, while a diet dominated by a single ingredient (e.g., plant proteins) may lead to disruption in microbial balance. Results have shown that carnivorous fish have a lower number of microbiota species compared with omnivorous fish [97].

Atlantic salmon (*Salmo salar*) fed a rapeseed meal-based diet had a lower total number of adherent bacteria in the intestine, as well as a more diverse population composition [90]. In black carp (*Mylopharyngodon piceus*) fed a diet in which up to 50% of the fishmeal was replaced by rapeseed meal, the dominant bacterial phyla in the intestinal microbiota were *Proteobacteria*, *Bacteroidetes*, and *Actinobacteria*. At the genus level, the dominant microbial genera in the intestinal flora were *Chrysobacillus*, *Achromobacillus*, and *Aeromonas* [80]. In one study [75], when a mixed meal containing rapeseed meal was compared with a control diet, the total aerobic bacteria decreased, while the total anaerobic bacteria increased. No differences were observed in *Escherichia coli*, *Aeromonas*, or *Bifidobacterium*.

## 6. Conclusions

The replacement of fishmeal and/or soybean meal with rapeseed meal in fish diets is the subject of numerous studies. Given the diversity within aquaculture, varying levels of rapeseed meal supplementation have been recorded for different fish species, leading to highly divergent results across individual studies. Generally, rapeseed meal has the potential to replace fishmeal and soybean meal to some extent. However, it is important to note that, in terms of digestive tract health, omnivorous fish show much greater tolerance for plant-based ingredients compared with carnivorous fish. Research on the impact of diet on some fish gut microbiota indicates that diets rich in rapeseed meal can alter the composition of the microbiota and cause an increase in the number of beneficial fiber-fermenting bacteria, while in other fish changes in the microbiota balance can lead to dysbiosis. The effects of rapeseed meal inclusion may vary depending on the fish species, rearing conditions, and other factors. In the future, it is recommended to conduct more research on the use of rapeseed meal in fish feed, particularly considering health aspects. Future studies should consider a detailed analysis of the gut microbiome, which has recently become a subject of interest for many scientists, especially since there are many scientific reports confirming numerous connections between health and production aspects and the composition of the gut microbiome. Knowledge about fish health, growth, gut microbial composition, and diversity can help improve aquaculture efficiency and reduce rearing costs. Future research should also focus on the application of technologies aimed at enhancing the nutritional value of rapeseed meal while concurrently reducing the levels of antinutritional factors. One promising approach involves the fermentation of rapeseed meal using naturally occurring microorganisms, which may improve nutrient availability and reduce antinutrient content. Moreover, the inclusion of feed additives such as exogenous enzymes—particularly phytase—should be further explored due to their proven ability to increase nutrient digestibility. Taking these activities into consideration could significantly enhance the potential of rapeseed meal as a dietary component, particularly for carnivorous fish species with high protein requirements.

## Figures and Tables

**Table 1:** Chemical composition [%], amino acid composition [g/100 g protein], minerals [%], and vitamin [mg/kg] content of rapeseed meal and soybean meal [38,39].

Chemical Composition [%]	Meal	
	Rapeseed	Soybean
Dry matter	88.0	88.0
Total protein	35.1	44.0
Crude fat	2.8	1.7
Crude fiber	12.6	6.4
Crude ash	6.6	6.4
Total carbohydrates	9.3	9.4
Essential amino acids [g/100 g protein]		
Lysine	5.8	6.4
Methionine	1.9	1.3
Phenylalanine	3.7	5.1
Histidine	2.7	2.6
Isoleucine	3.8	4.0
Leucine	6.6	7.8
Threonine	4.5	4.0
Tryptophan	1.3	1.4
Valine	5.2	4.9
Total	35.5	37.5
Non-essential amino acids [g/100 g protein]		
Tyrosine	2.5	3.2
Arginine	5.8	7.2
Alanine	4.3	4.3
Cysteine	2.4	1.6
Glycine	4.8	4.2
Aspartic acid	7.1	11.3
Glutamic acid	17.3	18.7
Proline	6.0	5.1
Serine	4.1	4.9
Total	54.3	60.5
Overall amino acids [g/100 g protein]	89.8	98.0
Minerals [%]		
Sodium	0.08	0.1
Potassium	1.17	2.0
Magnesium	0.6	0.3
Phosphorus	1.0	0.7
Calcium	0.7	0.3
Sulfur	0.7	0.4
Vitamins [mg/kg]		
Niacin	169.5	29.0
Riboflavin	3.7	2.9
Folic acid	2.3	1.3
Biotin	1.0	0.3
Thiamine	5.2	4.5
Panthotenic acid	9.5	16.0

**Table 2:** Effect of post-extraction rapeseed meal supplementation in fish feed as a protein source.

Species Studied	Initial Weight of Fish [g]	Experiment Duration [Weeks]	Supplementation Level of Rapeseed Meal	Significant Effect of Supplementation	References
European sea bass (*Dicentrarchus labrax*)	190	12	100 g/kg	No adverse effect on the growth rate, feed efficiency, or nitrogen utilization.	[71]
Hybrid sturgeon (*Acipenser baerii × Acipenser schrenckii*)	8.63	12	60–240 g/kg	Weight gain, specific growth rate, feed conversion ratio, and survival in fish fed the 240 g/kg rapeseed meal in diet were significantly lower than those in fish fed the control diet. Protein efficiency ratio in fish fed the 180 g/kg and 240 g/kg rapeseed meal in diet were significantly lower than those in fish fed the control diet.	[72]
Siberian sturgeon (*Acipenser baerii*)	22.80	10	10–40% substitution of fish meal	The lowest growth performance was observed in 40% substitution of fish meal. The results of the study showed that 30% fish meal can be replaced by rapeseed meal without negative effect on growth performance.	[67,73]
Siberian sturgeons (*Acipenser baerii*)	216.2	8	100–300 g/kg	No differences in all analyzed biometric and growth parameters were seen.	[61]
Red sea bream (*Pagrus major*)	4.5	8	285 g/kg	The final body weight, weight gain, specific growth rate, feed efficiency ratio, and protein efficiency were reduced; no effect on feed intake and survival.	[74]
Crucian carp (*Carassius auratus gibelio × Cyprinus carpio*)	21.9	8	500 g/kg without compensate for other nutrients	Reduction in the final body weight, growth rate, and protein efficiency ratio. Increased in feed conversion ratio.	[75]
Crucian carp (*Carassius auratus gibelio* × *Cyprinus carpio*)	21.9	8	500 g/kg with compensate for other nutrients	Reduced in protein efficiency ratio and increased in feed conversion ratio.	[75]
Nile tilapia (*Oreochromis niloticus*)	4.67	16	50–75% substitution of fish meal and soybean post-extraction meal	Reduced growth parameters at 75% substitution fish and soybean meal; no effect at 50% (the study results even suggested an improvement in growth performance).	[2]
Tilapia hybrids (*Oreochromis niloticus × Oreochromis aureus*)	6.8	8	30% substitution of soybean meal protein	Without significantly negatively affecting growth and body mass.	[76]
Yellow-headed catfish (*Pelteobagrus fulvidraco*)	2.38	10	Replacing 10% of fish meal with a mixture of plant meals (rapeseed and cottonseed meal in a 3:2 ratio)	Significant improvement in fish growth without negatively impacting their health.	[77]
Rainbow trout (*Oncorhynchus mykiss*)	17.2	12	32% crude protein	Diet did not affect the fish’s final live weight, daily weight gain, specific growth rate, feed conversion ratio, feed intake, and fish survival.	[78]
Rainbow trout (*Oncorhynchus mykiss*)	79.9	8	Up to 66% of fish meal replaced with rapeseed meal	Without a significant impact on growth parameters or the health status; all treatments at least tripled their weight.	[37]
Asian redtail catfish (*Hemibagrus wyckioides*)	3.24	8	11.20–44.80% share of feed	Rapeseed meal had no effect on fish survival. Feed intake decreased with increasing inclusion levels of rapeseed meal up to 11.2%, and then increased with further addition. Final body weight and weight gain declined with higher dietary levels of rapeseed meal. The protein efficiency ratio decreased, while the feed conversion ratio increased with rising levels of rapeseed meal in the diet.	[79]
Black carp (*Mylopharyngodon piceus*)	6.73	8	10–50% substitution of fish meal	Significantly reduced specific growth rate and weight gain above 20% replacement of fish meal with rapeseed.	[80]
Grass carp (*Ctenopharyngodon idellus*)	1.9	10	16–64% share of feed	Reduced feeding rate and specific growth rate at 64% share of rapeseed meal in feed; no effect at 16% share.	[81]
Nile tilapia (*Oreochromis niloticus*) and Mango tilapia (*Sarotherodon galilaeus*)	10.02	12	10–30% substitution of fish meal	Replacement of fish meal at a level of 20% resulted in increase in weight gain, length gain, weight gain rate, and specific growth rate; no effect of rapeseed on feed intake.	[62]
Japanese sea bass (*Lateolabrax japonicus*)	8.3	10	8.6–43.1% substitution of fish meal	Survival of fish significantly decreased with 40% dietary canola/rapeseed meal levels. Specific growth rate significantly decreased with 20% increasing dietary rapeseed levels.	[82]

## Data Availability

The original contributions presented in this study are included in the article. Further inquiries can be directed to the corresponding author.
